# Colistin and Isavuconazole Interact Synergistically In Vitro against *Aspergillus nidulans* and *Aspergillus niger*

**DOI:** 10.3390/microorganisms8091447

**Published:** 2020-09-21

**Authors:** Patrick Schwarz, Elie Djenontin, Eric Dannaoui

**Affiliations:** 1 Department of Internal Medicine, Respiratory and Critical Care Medicine, University Hospital Marburg, Baldingerstraße, D-35043 Marburg, Germany; 2 Center for Invasive Mycoses and Antifungals, School of Medicine, Philipps University Marburg, D-35037 Marburg, Germany; 3 Dynamyc Research Group (EA 7380), Faculté de Médecine de Créteil, Université Paris-Est-Créteil-Val-de-Marne, F-94010 Créteil, France; elie.djenontinagossou@aphp.fr (E.D.); eric.dannaoui@aphp.fr (E.D.); 4 Service de Microbiologie, Unité de Parasitologie-Mycologie, Hôpital Européen Georges-Pompidou, F-75015 Paris, France; 5 Faculté de Médecine, Université de Paris, F-75006 Paris, France

**Keywords:** antifungal resistance, antifungal combination, in vitro, aspergillosis, Aspergillus, cryptic species, isavuconazole, colistin, EUCAST

## Abstract

The in vitro interactions of isavuconazole in combination with colistin were evaluated against 55 clinical *Aspergillus* species isolates belonging to the five most important species (*Aspergillus flavus*, *Aspergillus fumigatus*, *Aspergillus nidulans*, *Aspergillus niger*, and *Aspergillus terreus*) responsible for human aspergillosis by a microdilution checkerboard technique based on the European Committee on Antimicrobial Susceptibility Testing (EUCAST) reference method for antifungal susceptibility testing. Selected isolates (*A. nidulans*, *n* = 10; *A. niger*, *n* = 15) were additionally evaluated by an agar diffusion assay using isavuconazole gradient concentration strips with or without colistin incorporated Roswell Parc Memorial Institute (RPMI) agar. Interpretation of the checkerboard results was done by the fractional inhibitory concentration index. Using the checkerboard method, combination isavuconazole–colistin was synergistic for 100% of the 15 *A. nidulans* isolates and for 60% of the 20 *A. niger* isolates. No interactions were found for any of the other isolates. By agar diffusion assay, minimal inhibitory concentrations (MICs) in combination decreased compared to isavuconazole alone for 92% of the isolates. No interactions were found for any *A. nidulans* isolates, but synergy was observed for 40% of the *A. niger* isolates. A poor essential agreement of EUCAST and gradient concentration strip MICs at ± 2 log_2_ dilutions with 0% was obtained. Antagonistic interactions were never observed regardless of the technique used.

## 1. Introduction

*Aspergillus* infections are an important problem in human health [1]. In immunocompromised patients, invasive aspergillosis mainly affects those with hematological malignancies, especially those with severe and prolonged neutropenia [2]. In non-immunocompromised patients with prior or current lung disease, chronic pulmonary aspergillosis can aggravate these preexisting lung diseases [3]. Another immunocompetent patient population at risk for *Aspergillus* infections comprise those with severe influenza. In a retrospective cohort study of seven intensive care units across Belgium and the Netherlands, 19% of 432 patients were diagnosed with invasive pulmonary aspergillosis [4]. Recently, the appearance of coronavirus disease 2019 (COVID-19), caused by severe acute respiratory syndrome coronavirus 2 [5], led to similar percentages of patients with invasive pulmonary aspergillosis as those known for severe influenza. In Cologne, 25% of patients with moderate to severe COVID-19-associated acute respiratory distress syndrome were diagnosed with invasive pulmonary aspergillosis. Similar percentages (19.4%) were reported from the Netherlands [6]. Species responsible for *Aspergillus* infections mainly include *Aspergillus fumigatus, Aspergillus flavus*, *Aspergillus terreus,* and *Aspergillus niger* [7]. Some of these species (e.g., *A. flavus*) are also able to produce harmful mycotoxins [8]. Recently, cryptic species, such as *Aspergillus lentulus,* were recognized [9]. Isolates of this species, formerly thought to be *A. fumigatus* isolates, showed low sporulation in multiple media and demonstrated decreased in vitro susceptibilities to multiple antifungals [10]. The treatment of choice for invasive aspergillosis is voriconazole [11], with isavuconazole recently expanding the portfolio of first-line treatments [12]. Nevertheless, azole-resistance represents an emerging problem, with primary resistance observed in several cryptic species [13] as well as acquired resistance in *A. fumigatus* [14], *A. terreus* [15,16], and *A. flavus* [17]. In *A. fumigatus,* acquired azole-resistance is mainly related to the TR34/L98H mutation in the *cyp51A* gene [18], which is also found in *A. fumigatus* isolates cultured from soil and compost, showing that the fungicides used in agriculture contribute to the emergence of this problem [19]. Antifungal combination may represent an interesting therapeutic approach to overcome this resistance, as this may decrease antifungal dosages and improve the pharmacokinetics of one or both drugs [20]. Another interesting approach to overcome azole-resistance is drug repurposing, with major advantages such as the use of de-risked compounds, with potentially lower overall development costs and shorter development timelines [21]. Colistin is a last-resort drug with activity against multidrug-resistant gram-negative bacteria such as *Pseudomonas aeruginosa*, *Klebsiella pneumoniae*, and *Acinetobacter baumanii*. [22] In *Candida albicans* [23] and *Rhizopus arrhizus* [24], colistin was shown to damage the cell membrane, making the molecule an interesting partner in combination therapy as demonstrated for *Candida auris*, where combination of colistin with azoles [25] or echinocandins [26] exhibited in vitro synergy. The aim of the present study is to evaluate the in vitro combination of colistin with isavuconazole against the five most important *Aspergillus* species responsible for human disease, including azole-resistant *A. fumigatus* isolates.

## 2. Materials and Methods

### 2.1. Isolates

Overall, 55 clinical *Aspergillus* species isolates, from the collection of the parasitology/mycology unit of Hôpital Européen Georges-Pompidou (HEGP), belonging to the 5 most important species responsible for human disease were used for the experiments. Isolates comprised 5 *A. flavus*, 10 *A. fumigatus,* 15 *A. nidulans*, 20 *A. niger*, and 5 *Aspergillus terreus*. All isolates were identified at the species level by sequencing a part of the beta-tubulin and/or calmodulin, as previously described [16]. Within the strains of the *A. nidulans* species complex, 14 *Aspergillus nidulans sensu stricto* and 1 *Aspergillus latus* were present. Within the strains of the *A. niger* species complex, 1 *Aspergillus luchuensis*, 2 *A. niger*, 1 *Aspergillus neoniger*, 8 *Aspergillus tubingensis*, and 8 *Aspergillus wellwitschiae* were present. The 10 *A. fumigatus* isolates included 5 azole-resistant strains (4 isolates with TR34/L98H alterations and 1 isolate with a G54W mutation). Before the experiments, isolates were subcultured from frozen stocks on Sabouraud dextrose agar slants supplemented with chloramphenicol and gentamycin (Bio-Rad Laboratories, Feldkirchen, Germany) for 7 days at 35 °C and 95% humidity to ensure purity and viability. The quality control reference strains *Candida krusei* (American Type Culture Collection) ATCC 6258 and *Candida parapsilosis* ATCC 22,019 were included in each series of experiments.

### 2.2. Medium Preparation

As the test medium, Roswell Park Memorial Institute (RPMI) 1640 medium (with L-glutamine and pH indicator but without bicarbonate) (Merck, Darmstadt, Germany) containing 2% D-Glucose (Merck), and buffered with 3-(N-morpholino)propanesulfonic acid (Merck) at a final concentration of 0.165 mol/L adjusted to pH 7.0 with 1 M sodium hydroxide (Merck) was used. To allow two-fold dilution, the test medium was prepared in double strength of the desired final concentration. After preparation, the medium was sterilized by vacuum filtration through a 0.22 µm pore-sized filter (Merck).

### 2.3. Drugs and Microplate Preparation

Combination studies were performed using the European Committee on Antimicrobial Susceptibility Testing (EUCAST) guidelines for antifungal susceptibility testing of molds with modifications for a broth microdilution checkerboard procedure. Experiments were carried out in Nunclon^TM^ delta surface 96-wells microtiter plates for adherent cells (Thermo Fisher Scientific, Darmstadt, Germany). The included drugs were isavuconazole (Pfizer, Berlin, Germany) and colistin (Merck). The stock solutions of isavuconazole (3200 µg/mL) and colistin (12,800 µg/mL) were prepared in dimethyl sulfoxide (DMSO) and sterile, distilled water, respectively. Drug dilutions were performed at four times the final concentration in double-strength RPMI medium. The combination was studied on a two-dimensional checkerboard with two-fold dilution. The final concentration for isavuconazole ranged from 0.03 to 16 µg/mL. The final concentration for colistin was 1 to 64 µg/mL. Fifty microliters of each concentration were distributed from row 1 to 8 for colistin and from column 1 to 11 for isavuconazole. Column 12 was used as growth control and contained 100 µL of double-strength RMPI medium with 1% DMSO.

### 2.4. Inoculum Preparation and Inoculation of Microplates

Before the preparation of the inoculum, all isolates were subcultured for a second time on Sabouraud dextrose agar slants containing chloramphenicol and gentamycin and incubated at 35 °C and 95% humidity. After incubation for 7 days, the spores were transferred to a sterile tube containing pure sterile water supplemented with 0.1% of Tween 80 (Merck) using a wet cotton swab. The suspension was counted in a hemocytometer and adjusted to 10^6^ conidia/mL. This solution was used to inoculate the RPMI agar plates. This solution was further diluted to 2 × 10^5^ conidia/mL with sterile water containing 0.1% Tween 80 in order to prevent growth of fungi on the surfaces of the wells [27]. One hundred microliters of the final inoculum was distributed in each well of the microplates. The final inoculum was further diluted and 100 µL was spread once on Sabouraud dextrose agar plates with a sterile Drigalski spatula. After 24–48 h of incubation at 35 °C, the colony-forming units were counted to ensure inoculum size and viability of the conidia. The microplates were incubated at 35 °C and 95% humidity, and the optical densities were read spectrophotometrically at 48 h at a wavelength of 530 nm using the spectrometer MultiSkan FC (Thermo Fisher Scientific). For each set of experiments, a blank microplate was used. Each well of the blank plates were inoculated with 100 µL of sterile distilled water containing 0.1% Tween 80. Blank microplates were also incubated at 35 °C and 95% humidity for 48 h. Before calculation of the MICs, the blank was subtracted from the inoculated microplates. All experiments were run in duplicate.

### 2.5. Interpretation of the Results

Minimal inhibitory concentrations (MICs) alone and in combination were determined as the lowest concentration giving complete inhibition as measured by 90% inhibition compared to the growth control by spectrophotometric reading. High off-scale MICs were converted to the next log_2_ dilution. The fractional inhibition concentration index (FICI) was interpreted as FICI ≤ 0.5 = synergy, FICI > 0.5–4 = no interaction, and FICI > 4.0 = antagonism [28].

### 2.6. Preparation of the RPMI Agar Plates

Fifteen grams of agar (Merck) were dissolved in 450 mL of distilled water. After adjustment of the pH to 7 with 0.1 M NaOH (Merck), the solution was set to 500 mL with distilled water and autoclaved for 20 min at 121 °C and 2 bars. The liquid agar and 500 mL of double-strength RPMI medium were put into a water bath. After temperature adjustment to 50 °C, the solutions were mixed under a flow bench and stirred using a stir fish. The colistin RPMI agar plates additionally contained an adequate volume of sterile colistin stock solution to a final concentration of 64 µg/mL for *A. niger* isolates and 32 µg/mL for *A. nidulans* isolates, as determined by preliminary experiments. In these preliminary experiments, 64 µg/mL was chosen for both species, but no growth was observed on the agar plates for colistin alone and in combination for any of the *A. nidulans* isolates. Therefore, 32 µg/mL was chosen for the *A. nidulans* isolates. Twenty microliters of the liquid RPMI agar or colistin RPMI agar was added to sterile Petri dishes (diameter 90 mm) (Merck) under a flow bench and allowed to cool down.

### 2.7. Inoculation of RPMI Agar Plates

The RPMI agar plates were inoculated using the inoculator retro C80 (bioMérieux, Nürtingen, Germany). Briefly, a sterile cotton swab was soaked in the spore suspension set to 10^6^ conidia/mL and excess fluid was removed by pressing the swab against the wall of the tube. A cross was gently drawn over the agar surface using the wet cotton swab. After the inoculator was turned on with maximum rotation, the cotton swab was moved from the edge of the agar surface to the middle of the plate and back while applying slight pressure. After inoculation, the plates were allowed to dry and isavuconazole gradient concentration strips (Liofilchem, Roseto degli Abruzzi, Italy) were placed onto the agar surface. For the activity of isavuconazole alone, plain RPMI agar plates were used. For the activity of colistin alone and for the combination, colistin RPMI agar plates were used. The activity of colistin alone was tested by inoculation of the agar plate without placement of a gradient concentration strip on the surface. Agar plates were incubated at 35 °C and pictures were taken at 48 h. MIC reading was done according to the recommendations of the manufacturer.

### 2.8. Interpretation of the Results of RPMI Agar Plates

A decrease or increase of ≥ 2 log_2_ dilutions in MIC compared to the most active drug was defined as synergy or antagonism, respectively. A decrease or increase of less than 2 log_2_ dilutions in MIC to the most active drug was defined as no interaction [29].

### 2.9. Comparison of EUCAST and Gradient Concentration Strip MICs

The essential agreement between the two techniques was evaluated by calculating the percentage of MICs, which differed by no more than 2log_2_ dilutions.

## 3. Results

In the first part of the experiments, the combination of isavuconazole with colistin was screened against 30 *Aspergillus* isolates belonging to five different species using the EUCAST broth microdilution technique. The results of these experiments are presented in Table 1. Based on these results, 25 additional (10 *A. nidulans* and 15 *A. niger*) isolates were tested using the EUCAST methodology and an agar diffusion assay. The results of these experiments are presented in Table 2. A summary of all results is presented in Table 3.

**Figure 1:**
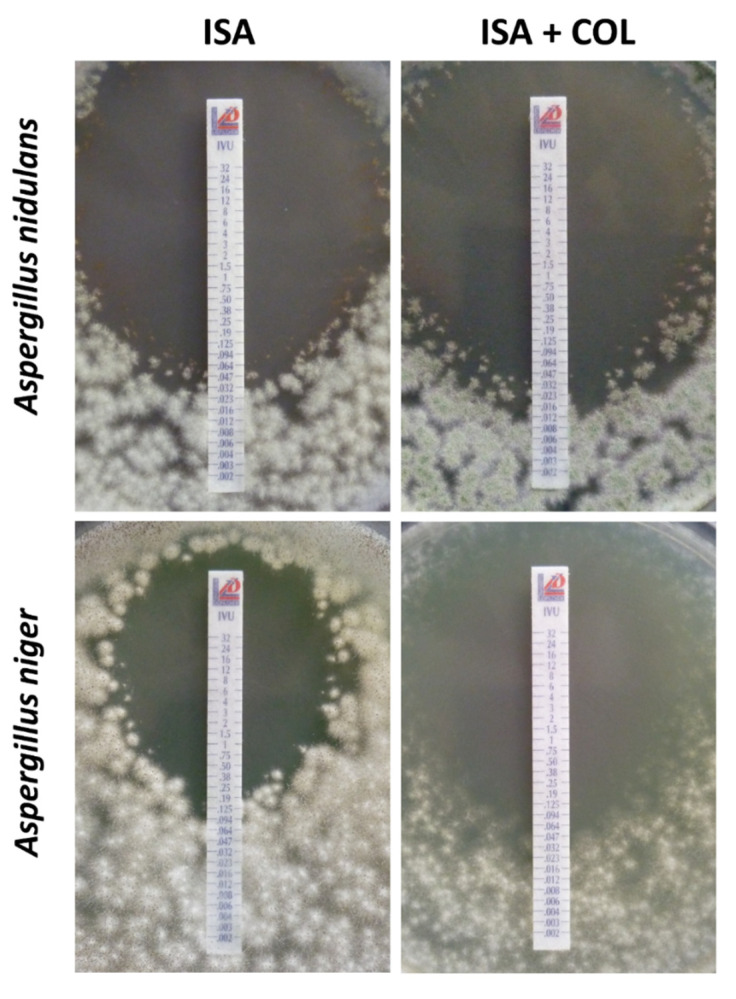
Interaction of isavuconazole and colistin vs. isavuconazole alone against *A. nidulans* isolate HEGP-3144 and *A. niger* isolate HEGP-6798 evaluated by agar diffusion assay. ISA, isavuconazole; COL, colistin; upper row, decrease of 1 log_2_ in combination compared to isavuconazole alone; lower row, synergistic interaction with decrease of 2 log_2_ in combination compared to isavuconazole alone.

The 55 isolates exhibited MICs for isavuconazole alone ranging from 0.5 to 16 μg/mL (Table 1 and Table 2) with MIC50, MIC90, and geometric mean MIC values of 2, 16, and 2.24 μg/mL, respectively. Isavuconazole MICs for *A. flavus*, *A. fumigatus*, *A. nidulans*, *A. niger*, and *A. terreus* ranged from 2 to 4 μg/mL, 1 to 16 μg/mL, 0.5 to 1 μg/mL, 4 to 16 μg/mL, and 0.5 to 1 μg/mL, respectively. Between experiments, isavuconazole MICs were within ± 1 log_2_ dilutions in 100% of the cases (data not shown). Colistin alone did not exhibit in vitro activity. MICs for all isolates were > 64 µg/mL. In the screening experiment, synergy was achieved for five of five *A. nidulans* isolates but only three of five *A. niger* isolates. For all other species, no interaction was obtained. To validate these results, an additional 10 *A. nidulans* and 15 *A. niger* isolates were tested. Using the checkerboard methodology, the combination exhibited synergy for 100% and 60% of the isolates of *Aspergillus* spp., respectively. For the *A. nidulans* isolates, die fractional inhibitory concentration index (FICI) ranged from 0.3125 to 0.5 with an average FICI of 0.39. For the *A. niger* isolates, die FICI ranged from 0.2813 to 0.625 with an average FICI of 0.48. According to the agar diffusion assay, MICs of isavuconazole for *A. nidulans* were 16 to 32-fold lower using the checkerboard method and ranged from 0.012 to 0.047 µg/mL, with MIC50, MIC90, and geometric mean MIC values of 0.016, 0.023, and 0.02 µg/mL, respectively. The correlation between EUCAST and gradient concentration strip MICs was poor, with an essential agreement at ± 2 log_2_ dilutions of 0%. There was a 3 to 5 log_2_ dilution difference for all *A. nidulans* isolates. Growth on RPMI agar was not inhibited by incorporation of 32 µg/mL of colistin for any *A. nidulans* isolates. Although all MICs in combination were lower than the MICs of isavuconazole alone, none of the isolates met the definition of synergy. Gradient concentration strip MICs of *A. niger* were also lower than those evaluated by checkerboard and ranged from 0.125 to 0.75 µg/mL, with MIC50, MIC90, and geometric mean MIC values of 0.38, 0.5, and 0.32 µg/mL, respectively. As for *A. nidulans*, the essential agreement between EUCAST and gradient concentration strip MICs for *A. niger* isolates at ± 2 log_2_ dilutions was 0%. The MIC dilution difference for all *A. niger* isolates was 3 to 5 log_2_. Colistin at 64 µg/mL incorporated in RMPI agar did not inhibit the growth of the tested *A. niger* isolates. Combination MICs were lower than MICs of isavuconazole alone for 13 of 15 isolates, with 6 isolates (40%) meeting the definition of synergy of a decrease ≥ 2 log_2_ dilutions compared to isavuconazole alone. A decrease of 1 log_2_ was seen for two additional isolates, whereas for two isolates the MICs in combination and for isavuconazole alone were the same. Figure 1 shows the interaction of isavuconazole and colistin against one *A. nidulans* and one *A. niger* isolate evaluated by agar diffusion assay. Antagonistic interactions were never observed, regardless off the in vitro technique used.

## 4. Discussion

The problems of primary and acquired azole-resistance in *Aspergillus* species are alarming [30], presenting an urgent need for new, broad-spectrum, antifungal drugs with new mechanisms of action [31]. However, strategies like combination therapy [20] or drug repurposing [21] could overcome this therapeutic issue. Therefore, we tested the combination of isavuconazole and colistin against a panel of both azole-susceptible and intrinsic or acquired azole-resistant *Aspergillus* species isolates.

The isavuconazole MICs of the tested *Aspergillus* isolates determined by ECUAST methodology in this study were in the same range as previously reported [32]. One drawback of EUCAST technique when testing molds is the visual determination of MICs. Previous reports demonstrated that spectrophotometric reading is a good alternative for *Aspergillus* spp. [33,34,35]. Incorporation of spectrophotometric reading leads to more objective MIC determination and automation of the process. Therefore, we chose spectrophotometric reading for MIC determination in this work.

The essential agreement between EUCAST and the gradient concentration strip MICs for *A. nidulans* and *A. niger* was poor. Two other studies evaluated the essential agreement between these two techniques for *A. niger* isolates, with one study, in contrast to ours, reporting an essential agreement of 93.75% [36]. These contrary results may be related to the different protocols (Clinical and Laboratory Standards Institute (CLSI) vs. EUCAST) used to perform the checkerboard experiments. The other study reported a poor essential agreement of 18.5% between the techniques using EUCAST methodology, similar to our results [37]. For colistin, we found no antifungal activity using the checkerboard method against *Aspergillus* species. These results were in accordance with previous studies [23,38].

Synergistic in vitro interactions between colistin and antifungal drugs were reported for yeasts [23,25,26,38,39,40] and filamentous fungi [23,38], as well as no interaction [23,26,40,41] and antagonism [41]. In this study, we found in vitro synergy for the combination of colistin with isavuconazole using the broth microdilution checkerboard technique for all tested *A. nidulans* isolates and 60% of the *A. niger* isolates. Colistin can induce cell membrane damage in *C. albicans* [23]. Azoles, which are inhibitors of ergosterol biosynthesis, also lead to alterations in the fungal membrane [42]. The synergistic effect of this combination may therefore be explained by the combined activity of the two drugs on the same cellular component. Using the agar diffusion assay, no interaction of the combination was seen for the *A. nidulans* isolates. For the *A. niger* isolates, synergy was seen in 40% of the isolates. Nevertheless, the combination MICs were reduced for all *A. nidulans* isolates and for 87% of the *A. niger* isolates compared to isavuconazole alone. The discrepancy between the results of the two techniques could be considered as evidence that the synergy of the combination is weak. Further evidence for weak synergy of the combination isavuconazole-colistin exists in the relatively high mean FICI values of 0.39 and 0.48 for *A. nidulans* and *A. niger*, respectively. To our knowledge, the combination of colistin with isavuconazole was previously only tested against *C. auris*, with a weak, but still synergistic, interaction with an average FICI of 0.4 reported [25]. Against *Aspergillus* species, only one study evaluated the in vitro interaction of colistin with antifungals. Interaction of liposomal amphotericin B with colistin exhibited synergy against three *A. fumigatus* isolates [38]. In our study, the combination of colistin with isavuconazole was inactive against *A. fumigatus*.

Concerning the potential usability of the combination in patients, peak serum levels of 13 to 32 µg/mL were reported in patients with cystic fibrosis receiving prolonged colistin therapy [43]. These concentrations would be sufficient, as in our study we found MICs of colistin in combination of 8 to 32 μg/mL and 1 to 32 µg/mL for *A. nidulans* and *A. niger*, respectively. Nevertheless, due to its nephrotoxicity [44], the use of colistin is limited in critically ill patients and serum peak levels are lower (0.5 to 9.4 µg/mL) [45]. However, it cannot be excluded that in vivo synergy can be present at lower concentrations than those evaluated in vitro, as demonstrated for the combination of tacrolimus with fluconazole or amphotericin in patients with cryptococcosis [46]. For isavuconazole, mean blood concentrations of 3.6 µg/mL (range 0.64–8.13 µg/mL) were measured in patients during the first 30 days of treatment [47], which would be sufficient as we found ranges of 0.03 to 0.25 μg/mL and 1 to 4 µg/mL (geometric mean 2.38 µg/mL) MICs in combination for *A. nidulans* and *A. niger*, respectively.

In summary, colistin enhances the in vitro activity of isavuconazole against clinical *A. nidulans* isolates and *A. niger* isolates with high in vitro MICs to isavuconazole. These results warrant further in vivo experiments.

## Figures and Tables

**Table 1:** In vitro screening of isavuconazole in combination with colistin against *Aspergillus* species using EUCAST broth microdilution checkerboard methodology.

Species	Collection Number	MIC (µg/mL)				
		ISA	COL	ISA/COL	FICI	INTPN
*A. flavus*	HEGP-6097	4	128	2/64	1	IND
*A. flavus*	HEGP-5899	4	128	4/1	1.0078	IND
*A. flavus*	HEGP-4536	2	128	2/2	1.0156	IND
*A. flavus*	HEGP-4251	2	128	2/1	1.0078	IND
*A. flavus*	HEGP-4114	2	128	2/2	1.0156	IND
*A. fumigatus*	HEGP-5780 ^(a)^	16	128	8/64	1	IND
*A. fumigatus*	HEGP-4020 ^(b)^	1	128	1/2	1.0156	IND
*A. fumigatus*	HEGP-4083 ^(a)^	16	128	16/2	1.0156	IND
*A. fumigatus*	HEGP-2659 ^(a)^	16	128	8/64	1	IND
*A. fumigatus*	HEGP-2664 ^(a)^	8	128	8/1	1.0078	IND
*A. fumigatus*	HEGP-R117	1	128	0.5/64	1	IND
*A. fumigatus*	HEGP-R279	1	128	1/1	1.0078	IND
*A. fumigatus*	HEGP-R285	1	128	0.5/64	1	IND
*A. fumigatus*	HEGP-R290	2	128	1/64	1	IND
*A. fumigatus*	HEGP-R291	1	128	0.5/64	1	IND
*A. nidulans*	HEGP-5711 ^(c)^	0.5	128	0.12/16	0.375	SYN
*A. nidulans*	HEGP-6169 ^(c)^	0.5	128	0.06/32	0.375	SYN
*A. nidulans*	HEGP-5492 ^(c)^	0.5	128	0.12/32	0.5	SYN
*A. nidulans*	HEGP-5521 ^(d)^	0.5	128	0.12/16	0.375	SYN
*A. nidulans*	HEGP-5329 ^(c)^	0.5	128	0.03/32	0.3125	SYN
*A. niger*	HEGP-6071 ^(e)^	8	128	2/32	0.5	SYN
*A. niger*	HEGP-6217 ^(f)^	8	128	2/32	0.5	SYN
*A. niger*	HEGP-6475 ^(g)^	8	128	4/16	0.625	IND
*A. niger*	HEGP-6562 ^(e)^	8	128	2/32	0.5	SYN
*A. niger*	HEGP-6917 ^(g)^	4	128	2/16	0.625	IND
*A. terreus*	HEGP-6625	1	128	1/1	1.0078	IND
*A. terreus*	HEGP-6055	1	128	1/1	1.0078	IND
*A. terreus*	HEGP-5599	0.5	128	0.5/1	1.0078	IND
*A. terreus*	HEGP-5169	0.5	128	0.5/1	1.0078	IND
*A. terreus*	HEGP-6398	0.5	128	0.25/2	0.5156	IND

SYN, synergy (FICI ≤ 0.5); IND, no interaction (0.5 < FICI ≤ 4); ISA, isavuconazole; COL, colistin; FICI, fractional inhibitory concentration index; INTPN, interpretation; HEGP, Hôpital Européen Georges-Pompidou. ^(a)^ isolate with TR34/L98H alteration; ^(b)^ isolate with a G54W mutation. Within the strains of the *A. nidulans* species complex, there were four *A. nidulans sensu stricto* ^(c)^ and one *A. latus* ^(d)^. Within the strains the of *A. niger* species complex, there were two *A. tubingensis* ^(e)^, one *A. luchuensis* ^(f)^, and two *A. wellwitschiae* ^(g)^.

**Table 2:** In vitro combination of isavuconazole with colistin against *Aspergillus nidulans* and *Aspergillus niger* isolates using EUCAST broth microdilution checkerboard methodology and agar diffusion assay.

Species	Collection Number	Checkerboard MICs (µg/mL)					Agar Diffusion Assay MICs (µg/mL)		
		ISA	COL	ISA/COL	FICI	INTPN ^a^	ISA	ISA + COL	INTPN ^b^
*A. nidulans*	HEGP-2971	1	128	0.25/8	0.3125	SYN	0.023	0.012	IND
*A. nidulans*	HEGP-3143	0.5	128	0.06/32	0.375	SYN	0.016	0.016	IND
*A. nidulans*	HEGP-3144	1	128	0.25/16	0.375	SYN	0.047	0.023	IND
*A. nidulans*	HEGP-3906	0.5	128	0.12/32	0.5	SYN	0.012	0.008	IND
*A. nidulans*	HEGP-4034	0.5	128	0.12/8	0.3125	SYN	0.016	0.006	IND
*A. nidulans*	HEGP-4149	0.5	128	0.12/16	0.375	SYN	0.032	0.016	IND
*A. nidulans*	HEGP-1520	0.5	128	0.12/32	0.5	SYN	0.023	0.008	IND
*A. nidulans*	HEGP-1874	0.5	128	0.12/16	0.375	SYN	0.016	0.012	IND
*A. nidulans*	HEGP-2290	0.5	128	0.06/32	0.375	SYN	0.016	0.008	IND
*A. nidulans*	HEGP-2473	0.5	128	0.12/16	0.375	SYN	0.016	0.008	IND
*A. niger*	HEGP-6029 ^(a)^	8	128	4/2	0.5156	IND	0.38	0.25	IND
*A. niger*	HEGP-6036 ^(c)^	8	128	4/8	0.5625	IND	0.38	0.19	IND
*A. niger*	HEGP-6678 ^(b)^	8	128	4/4	0.5313	IND	0.38	0.25	IND
*A. niger*	HEGP-6760 ^(d)^	8	128	2/16	0.375	SYN	0.38	0.19	IND
*A. niger*	HEGP-6785 ^(c)^	8	128	4/16	0.625	IND	0.38	0.047	SYN
*A. niger*	HEGP-6798 ^(d)^	4	128	1/4	0.2813	SYN	0.19	0.047	SYN
*A. niger*	HEGP-7157 ^(d)^	8	128	2/16	0.375	SYN	0.25	0.19	IND
*A. niger*	HEGP-7304 ^(d)^	4	128	1/16	0.375	SYN	0.38	0.032	SYN
*A. niger*	HEGP-7312 ^(c)^	4	128	2/4	0.5313	IND	0.19	0.19	IND
*A. niger*	HEGP-7313 ^(d)^	4	128	2/1	0.5078	IND	0.125	0.094	IND
*A. niger*	HEGP-7460 ^(d)^	4	128	1/16	0.375	SYN	0.38	0.094	SYN
*A. niger*	HEGP-7467 ^(a)^	8	128	2/32	0.5	SYN	0.19	0.125	IND
*A. niger*	HEGP-7501 ^(c)^	16	128	4/16	0.375	SYN	0.5	0.125	SYN
*A. niger*	HEGP-7511 ^(c)^	16	128	4/32	0.5	SYN	0.5	0.5	IND
*A. niger*	HEGP-7546 ^(c)^	16	128	4/16	0.375	SYN	0.75	0.125	SYN

^a^ SYN, synergy (FICI ≤ 0.5); IND, no interaction (0.5 < FICI ≤ 4). ^b^ SYN, synergy (decrease ≥2 log_2_ dilutions in MIC compared to the most active drug); IND, no interaction (decrease or increase <2 log_2_ dilutions in MIC compared to the most active drug. ISA, isavuconazole; COL, colistin; FICI, fractional inhibitory concentration index; INTPN, interpretation; HEGP, Hôpital Européen Georges-Pompidou. Within the strains of the *A. niger* species complex, there were two *A. niger* ^(a)^, 1 *A. neoniger* ^(b)^, six *A. tubingensis* ^(c)^, and six *A. wellwitschiae* ^(d)^. All *A. nidulans* species complex isolates were *A. nidulans sensu stricto*.

**Table 3:** Summary of in vitro interactions of isavuconazole with colistin against *Aspergillus* species using EUCAST broth microdilution checkerboard methodology and agar diffusion assay.

Species (Isolates), Technique	% of Isolates with the Following Interaction		
	Synergy	No Interaction	Antagonism
*A. flavus* (5), EUCAST	0	100	0
*A. fumigatus* (10), EUCAST	0	100	0
*A. nidulans* (15), EUCAST	100	0	0
*A. nidulans* (10), ADA	0	100	0
*A. niger* (20), EUCAST	60	40	0
*A. niger* (15), ADA	40	60	0
*A. terreus* (5), EUCAST	0	100	0

ADA, agar diffusion assay.

## Congress Presentations

Part of the results of this work (abstract number P012) were presented at the 9th Trends in Medical Mycology, 11–14 October 2019 in Nice, France.
